# Real-time PCR assay and rapid diagnostic tests for the diagnosis of clinically suspected malaria patients in Bangladesh

**DOI:** 10.1186/1475-2875-10-175

**Published:** 2011-06-26

**Authors:** Mohammad Shafiul Alam, Abu Naser Mohon, Shariar Mustafa, Wasif Ali Khan, Nazrul Islam, Mohammad Jahirul Karim, Hamida Khanum, David J Sullivan, Rashidul Haque

**Affiliations:** 1 Parasitology Laboratory, ICDDR,B, GPO Box 128, Dhaka-1000, Bangladesh; 2 Malaria and Parasitic Disease Control Unit, Directorate General of Health Services, Mohakhali, Dhaka 1212, Bangladesh; 3 Department of Zoology, University of Dhaka, Dhaka 1000, Bangladesh; 4 Johns Hopkins Malaria Research Institute, Bloomberg School of Public Health, Baltimore, MD, USA

## Abstract

**Background:**

More than 95% of total malaria cases in Bangladesh are reported from the 13 high endemic districts. *Plasmodium falciparum *and *Plasmodium vivax *are the two most abundant malaria parasites in the country. To improve the detection and management of malaria patients, the National Malaria Control Programme (NMCP) has been using rapid diagnostic test (RDT) in the endemic areas. A study was conducted to establish a SYBR Green-based modified real-time PCR assay as a gold standard to evaluate the performance of four commercially-available malaria RDTs, along with the classical gold standard- microscopy.

**Methods:**

Blood samples were collected from 338 febrile patients referred for the diagnosis of malaria by the attending physician at Matiranga

Upazila Health Complex (UHC) from May 2009 to August 2010. Paracheck RDT and microscopy were performed at the UHC. The blood samples were preserved in EDTA tubes. A SYBR Green-based real-time PCR assay was performed and evaluated. The performances of the remaining three RDTs (Falcivax, Onsite Pf and Onsite Pf/Pv) were also evaluated against microscopy and real-time PCR using the stored blood samples.

**Result:**

In total, 338 febrile patients were enrolled in the study. Malaria parasites were detected in 189 (55.9%) and 188 (55.6%) patients by microscopy and real-time PCR respectively. Among the RDTs, the highest sensitivity for the detection of *P. falciparum *(including mixed infection) was obtained by Paracheck [98.8%, 95% confidence interval (CI) 95.8-99.9] and Falcivax (97.6%, 95% CI 94.1-99.4) compared to microscopy and real-time PCR respectively. Paracheck and Onsite Pf/Pv gave the highest specificity (98.8%, 95% CI 95.7-99.9) compared to microscopy and Onsite Pf/Pv (98.8, 95% CI 95.8-99.9) compared to real-time PCR respectively for the detection of *P. falciparum*. On the other hand Falcivax and Onsite Pf/Pv had equal sensitivity (90.5%, 95% CI 69.6-98.8) and almost 100% specificity compared to microscopy for the detection of *P. vivax*. However, compared to real-time PCR assay RDTs and microscopy gave low sensitivity (76.9%, 95% CI 56.4-91) in detecting of *P. vivax *although a very high specificity was obtained (99- 100%).

**Conclusion:**

The results of this study suggest that the SYBR Green-based real-time PCR assay could be used as an alternative gold standard method in a reference setting. Commercially-available RDTs used in the study are quite sensitive and specific in detecting *P. falciparum*, although their sensitivity in detecting *P. vivax *was not satisfactory compared to the real-time PCR assay.

## Background

Malaria is still considered a major public-health problem in the eastern districts of Bangladesh, bordering India and Myanmar. These districts experience a perennial transmission of malaria with two peaks in pre-monsoon (March-May) and post-monsoon (September-November) periods [1]. In the changing climatic situation and in absence of major malaria vectors, such as *Anopheles minimus *and *Anopheles baimaii *a number of *Anopheles *species have been incriminated and playing a role in the transmission of malaria in the country [2]. *Plasmodium falciparum *and *Plasmodium vivax *are two main malaria parasites in the country as reported by a nationwide prevalence survey in 2007. The survey showed that contribution of *P. falciparum *was 90.18%, followed by *P. vivax *(5.29%), and the remaining (4.53%) was mixed infection of these two species [3].

The Giemsa-stained blood slide using thin and thick smears for malaria parasites has been the gold standard method for nearly a century [4]. No alternative method still could be established to replace this universally-accepted gold standard method. Such a laboratory technique to confirm the clinical suspicion of malaria is labour-intensive [5] and sometimes unreliable due to lack of skilled microscopists, limited supplies, inadequate maintenance of microscopes and reagents, and inadequate or absence of quality-control systems [6].

In recent time, lateral flow immunochromatographic-based rapid diagnostic test (RDT) has been developed for the diagnosis of suspected malaria patients and are widely used in remote areas across the world [7]. Most RDTs are intended to react with antigens commonly released from or enzymes present in parasitized red blood cells. In the case of *P. falciparum*, the water soluble histidine-rich protein-2 (HRP-2) antigen is commonly used as it is specific to *P. falciparum *associated infection. Non-falciparum malaria or mixed infections with *P. falciparum *are commonly detected by *Plasmodium *lactate dehydrogenase (pLDH) [8,9]. In the Global Fund sponsored malaria control programme RDT is recommended and being widely used for detecting malaria cases in the endemic areas of Bangladesh [1].

The molecular detection method, such as polymerase chain reaction (PCR) has been developed to diagnose *Plasmodium *spp. and has been performed in several places for routine diagnosis or for evaluating the performance of microscopy or RDT [10-14]. In recent time, real-time PCR method has been established for the quantitative detection of malaria parasites [15-19]. Real-time PCR is reliable and yield high sensitivity and specificity when compared with microcopy or nested PCR [15,19,20].

This study demonstrated a SYBR Green-based modified real-time method to use it as a gold standard, along with conventional microscopy to evaluate four RDTs for diagnosis of malaria from suspected febrile patients. Such a study has never been done before in Bangladesh. The study would provide additional support to the NMCP for monitoring and evaluation of the performances of the diagnostic methods used in their ongoing malaria control programme.

## Methods

### Study area and population

The study was conducted at Matiranga Upazila (sub-district) of Khagrachari district situated at the south-eastern part of Bangladesh. Febrile patients referred to microscopy for malaria diagnosis at Matiranga Upazila Health Complex (UHC) from May 2009 to August 2010 were enrolled. The recent malaria prevalence survey, Matiranga showed high prevalence of asymptomatic malaria cases (21.6%) [21].

### Sample collection

Five ml of blood was taken from an adult subject and in case of children or minor subjects three ml of blood was obtained through venipuncture by an experienced medical technologist. Two drops of sample were used for preparing thick and thin smear slides, one drop was used for Paracheck RDT, and the remaining samples were preserved in an EDTA tube and stored at -20°C.

### Microscopy

The blood film was stained with Giemsa in phosphate buffer saline and examined under the compound microscope at a magnification of ×1,000 for malaria parasites. Blood films were defined as negative if no parasite was observed in 100× oil immersion fields (magnification, ×1,000) on thin film by an experienced microscopist [22]. Declaring a slide positive or negative and initial speciation was routinely based on the examination of 200 fields in the Giemsa-stained thick film. A slide was considered positive when at least one parasite was found. After finding the first parasite, another 200 fields were completed for any mixed infection. If no parasite was found in 200 oil fields, the slide was considered negative. Density of the parasite was measured from thick blood smears by counting the number of parasites per 200 leukocytes and expressed as parasites/μl. In the case of 10 or less parasites, 500 leukocytes were counted. Each slide was assessed by two independent microscopists; one of them was employed by the study and the other person was posted at Matiranga UHC. A slide was considered positive only when these two microscopists were in agreement. There was a provision for third microscopist posted at the Khagrachari Civil Surgeon's office situated 20 km away from Matiranga UHC for any disagreement between them.

### Rapid diagnostic tests

In the present investigation four RDTs (device) were used. These were Paracheck (Orchid Biomedical System, India), FalciVax Pf (Zephyr Biomedicals, India), Onsite Pf (CTK Biotech Inc, USA) and Onsite Pf/Pv (CTK Biotech Inc, USA). Paracheck and Onsite Pf used *P. falciparum*-specific HRP-2 antigen. FalciVax and Onsite Pf/Pv used *P. vivax*- specific pLDH together with *P. falciparum*-specific HRP-2.

All the RDTs were used following the instructions of the manufacturers. 'Paracheck' is being used by the National Malaria Control Programme (NMCP) in the endemic areas and was available at the MUHC. Paracheck test was performed at Matiranga UHC concurrently with the microscopy. The remaining three RDTs were performed using the stored samples as per the instructions of the manufacturers.

### DNA extraction

DNA was extracted from 200 μl EDTA preserved blood samples using the QiaAmp blood mini kit (QIAGEN, Inc., Germany) following the manufacturer's instructions at the Parasitology Laboratory of ICDDR,B. DNA sample was stored at 4°C until PCR could be completed.

### Real-time PCR

Real-time PCR was done by the primer sets described by Perandin *et al *[15] with some modification to a single-plex reaction. Instead of TaqMan probe, SYBR Green I dye was used for visualizing the amplification. PCR condition was also modified slightly to fit with Platinum^® ^SYBR^® ^Green qPCR SuperMix-UDG (Invitrogen Corporation, USA) following the instructions of the manufacturer. Purified DNA templates were amplified in a BioRad CFX-96 real time system (BioRad, USA) with a species-specific primer set. Briefly, a 25-μl PCR mixture was prepared using 1 μl of template DNA, 12.5 μl Platinum SYBR Green qPCR supermix (PlatinumR *Taq *DNA polymerase, SYBR Green I dye, Tris-HCl, KCl, 6 mM MgCl2, 400 μM dGTP, 400 μM dATP, 400 μM dCTP, 800 μM dUTP, uracil DNA glycosylase, and stabilizers), 320 nM concentration of each of parasite species-specific primer set. Amplification and detection were performed as follows: 50°C for 2 min and 95°C for 2 min. After that 95°C for 1 min, 58°C for 1 min and 72°C for 1 min 30 sec for a single cycle were performed. 40 cycles were considered for *P. falciparum *and 35 cycles for *P. vivax*. The plate read was taken after the extension at 72°C. The melt curve was prepared from 50°C to 95°C with an increment of 0.5°C each after five seconds.

To establish the minimum number of parasites detectable by the *Plasmodium *SYBR Green assay (detection limit), blood samples from two patients infected, respectively, with *P. falciparum *(one patient) and *P. vivax *(one patient) were collected, and parasitaemia was calculated using 200 WBC count as reference. The infected blood samples were diluted with uninfected erythrocytes from healthy individuals with known baseline erythrocyte counts. Ten-fold serial dilution was made to obtain a final parasitaemia of 1% (1 parasite/μl of blood) for each sample. All DNA aliquots purified from the dilutions were treated in duplicate for real-time PCR assay. To estimate the analytical specificity of the *Plasmodium *real-time PCR assay, DNA from *in vitro *culture samples of other protozoan parasites, such as *Entamoeba histolytica *and *Leishmania donovani *were used. The clinical sensitivity and specificity of the modified *Plasmodium *real-time PCR assay for detecting and identifying malaria parasites were calculated on 338 whole-blood samples, microscopy as the gold standard and vice- versa.

### Analysis of data

The performance of each method was calculated by means of sensitivity, specificity, positive predictive value (PPV), and negative predictive value (NPV) using microscopy and modified real-time PCR as gold standard. SPSS software version 11.5 (SPSS Inc., USA) was used for calculating the kappa coefficient (k) of the tests for each association using the X^2 ^test. Sensitivity, specificity, positive predictive value and negative predictive value were calculated using the 'diagt' command of the STATA software version 10 (Stata Corp, USA)[23].

### Ethical approval

The study was approved by the Research Review Committee and Ethical Review Committee of ICDDR,B. Approval was also obtained from the NMCP for the study. Informed consent was obtained from all adult subjects, and assent was obtained from the legal guardians in the case of minor subjects before the collection of blood sample. Good clinical and laboratory practices were followed in all the procedures.

## Results

### Enrollment

In total, 338 febrile patients were recruited for the study from May 2009 to August 2010. Of them, 50.3% were female. The age of the patients ranged from 18 months to 82 years, with a median age of 14 years.

### Microscopy

Malaria parasites were detected in 189 (55.9%) patients by microscopy. Of them 168 (88.9%) were infected by *P. falciparum*, 18 (9.5%) patients by *P. vivax *and remaining three (1.6%) patients had a mixed infection of *P. falciparum *and *P. vivax *(Table 1). Overall, high parasite count was observed in microscopy. Parasite count ranged from 16 to 261,480 parasites/μl of blood. A median number of parasite count of 19,960 [interquartile range (IQR) 6,280-48,320] parasites/μl of blood was found in 171 *P. falciparum *positive patients. Only six (3.5%) of the samples were below 100 parasites/ μl whereas 118 (69%) had a count of more than 10,000 parasites/ μl of blood. Of 21 *P. vivax *positive slides, the parasite count ranged from 32 to 25,120 parasites/μl of blood, with a median of 5,040 (IQR 520-17,160) parasites/μl of blood. One (4.8%) sample had a parasite count below 100 parasites/ μl whereas 15 (71.4%) of the sample had a count of more than 1,000 parasites/μl of blood (see Additional file 1).

**Table 1:** Results for different tests used in the study

Test	Negative	Positive			
		
	N (%)	Pf (%)	Pv (%)	Pf + Pv mixed (%)	Total (%)
Microscopy	149 (44.1)	168 (49.7)	18 (5.3)	3 (0.9)	189(55.9)
Paracheck	167 (49.4)	171 (50.6)	N/A	N/A	171 (50.6)
Onsite Pf	174 (51.5)	164(48.5)	N/A	N/A	164(48.5)
Falcivax	147 (43.5)	171 (50.6)	18 (5.3)	2 (0.6)	191 (56.5)
Onsite Pf/Pv	160 (47.3)	155 (45.9)	18 (5.3)	5 (1.5)	178 (52.7)
Real-time PCR	150 (44.4)	162 (47.9)	18 (5.3)	8 (2.4)	188(55.6)

### Real-time PCR

**Figure 1:**
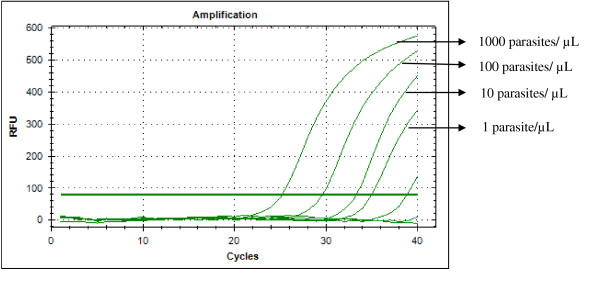
**Typical amplification curve (generated by CFX-96 Real-Time System) for *P. falciparum***.

**Figure 2:**
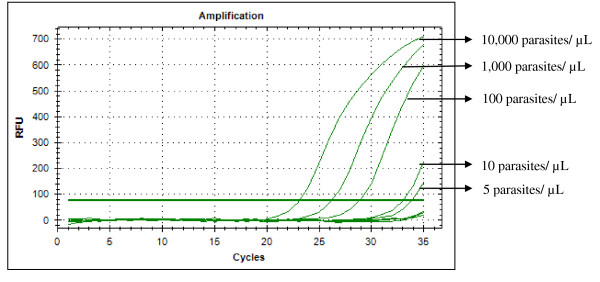
**Typical amplification curve (generated by CFX-96 Real-Time System) for *P. vivax***.

**Figure 3:**
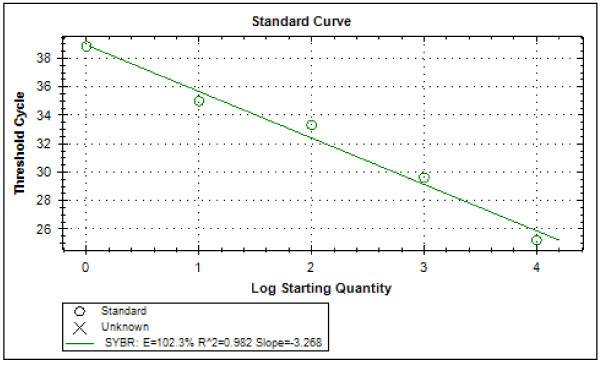
**Standard curve for *P. falciparum *produced against CT values and logarithm of parasite count/μL of blood (R^2 ^= 0.982)**.

**Figure 4:**
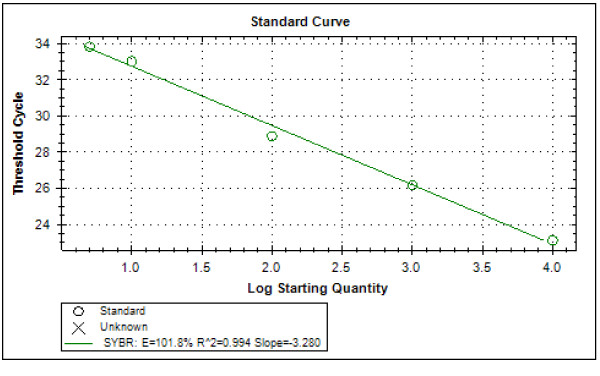
**Standard curve for *P. vivax *produced against CT values and logarithm of parasite count/μL of blood (R^2 ^= 0.994)**.

**Figure 5:**
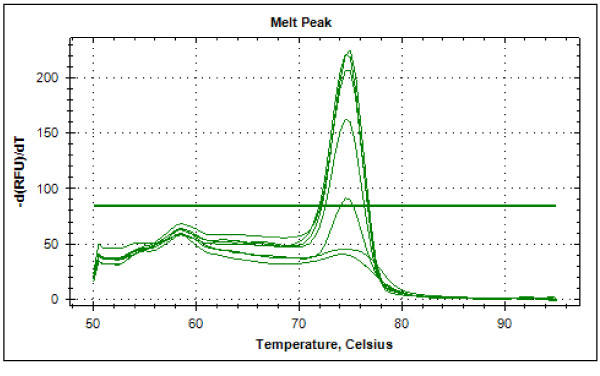
**Typical melt curve of *P. falciparum *showing peak at 74.5°C**.

**Figure 6:**
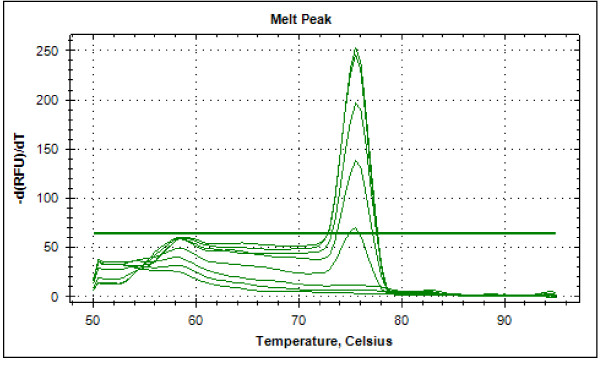
**Typical melt curve of *P. vivax *showing peak at 75.5°C**.

Typical displays (amplification plots) for *P. falciparum *and *P. vivax *by the SYBR Green I PCR assay provided by Bio Rad CFX-96 are shown in Figures 1 and 2. Positive signals by means of cycle threshold [CT] value were obtained for all dilutions, with a detection limit of 5-10 parasites/μl for *P. falciparum *and *P. vivax *in different experiments. Reproducible linearity of over a 10,000-fold range was shown by CT values. A significant correlation coefficient was found for the mean CT values and parasitaemia (*P. falciparum*, R^2 ^= 0.982; *P. vivax*, R^2 ^= 0.994) (Figures 3 and 4). For non-*Plasmodium *protozoan DNA (*E. histolytica *and *L. donovani*) and blood DNA samples of healthy human subjects no signal was obtained by the SYBR Green real-time PCR. The melt peak for *P. falciparum and P. vivax *was found at 74.5°C and 75.5°C from the corresponding positive controls respectively (Figures 5 and 6). Any amplification other than these two melting temperatures was excluded as false amplification.

Using the real-time PCR assay results 188 (55.6%) samples were found positive for any malarial infection (Table 1). Of the 188 PCR positive samples 162 (86.2%) were infected by *P. falciparum*, 18 (9.5%) were infected by *P. vivax *and the remaining 8 (4.3%) samples were mixed infection with *P. falciparum and P. vivax *(Table 1). Sensitivity, specificity, positive predictive value, negative predictive value, and kappa (k) of PCR assay compared to microscopy are given in table 2. For the detection of *P. falciparum *(including mixed infection), modified real-time PCR assay had 97.1% (95% CI: 93.3-99) sensitivity and 97.6% (95% CI: 94-99.3) specificity respectively. While for the detection of *P. vivax *(including mixed infection) modified real-time PCR showed 95.2% (95% CI: 76.2-99.9) sensitivity and 98.1% (95% CI: 95.9-99.6) specificity respectively (Table 2).

**Table 2:** Sensitivity, specificity, positive predictive value, and negative predictive value of RDTs and real-time PCR versus microscopy as gold standard

Method	Test	Sensitivity (95% CI)	Specificity (95% CI)	PPV (95% CI)	NPV (95%CI)	Kappa (k)
Paracheck	Pure and mixed Pf	98.8 (95.8-99.9)	98.8 (95.7-99.9)	98.8 (95.8-99.9)	98.8 (95.7-99.8)	0.98
Falcivax	Pure and mixed Pf	98.2 (95-99.6)	97 (93.2-99)	97.1 (93.4-99.1)	98.2 (94.8-99.6)	0.95
Onsite Pf	Pure and mixed Pf	93.6 (88.8-96.7)	97.6 (94-99.3)	97.6 (93.9-99.3)	93.7 (89-96.8)	0.91
Onsite Pf/Pv	Pure and mixed Pf	92.4 (87.4-95.9)	98.8 (95.7-99.9)	98.8 (95.6-99.8)	92.7 (87.8-96.1)	0.91
PCR	Pure and mixed Pf	97.1 (93.3-99)	97.6 (94-99.3)	97.6 (94.1-99.4)	97 (93.2-99)	0.95
Falcivax	Pure and mixed Pv	90.5 (69.6-98.8)	99.7 (98.3-100)	95 (75.1-99.9)	99.4 (97.7-99.9)	0.92
Onsite Pf/Pv	Pure and mixed Pv	90.5 (69.6-98.8)	98.7 (96.8-99.7)	82.6 (61.2-95)	99.4 (97.7-99.9)	0.85
PCR	Pure and mixed Pv	95.2 (76.2-99.9)	98.1 (95.9-99.6)	76.9 (56.4-91)	99.7 (98.2-100)	0.84

(PPV = Positive predictive value, NPV = Negative predictive value)

Compared to real-time PCR assay, microscopy had 97.6% sensitivity (95% CI: 94.1-99.4) and 97% (95%CI 93.2-99) specificity for the detection of *P. falciparum *and 76.9% sensitivity (95% CI: 56.4-91) and 99.7% specificity (95% CI: 98.2-100) for the detection of *P. vivax *respectively (Table 3).

**Table 3:** Sensitivity, specificity, positive predictive value and negative predictive value of RDTs and microscopy versus real-time PCR as gold standard

Method	Test	Sensitivity (95% CI)	Specificity (95% CI)	PPV (95% CI)	NPV (95%CI)	Kappa (k)
Paracheck	Pure and mixed Pf	97.1 (93.3-99)	96.4 (92.4-98.7)	96.5 (92.5-98.7)	97 (93.2-99)	0.94
Falcivax	Pure and mixed Pf	97.6 (94.1-99.4)	95.8 (91.6-98.3)	96 (91.8-98.4)	97.6 (93.9-99.3)	0.94
Onsite Pf	Pure and mixed Pf	94.1 (89.4-97.1)	97.6 (94-99.3)	97.6 (93.9-99.3)	94.3 (89.7-97.2)	0.92
Onsite Pf/Pv	Pure and mixed Pf	92.9 (88-96.3)	98.8 (95.8-99.9)	98.8 (95.6-99.8)	93.3 (88.5-96.5)	0.92
Microscopy	Pure and mixed Pf	97.6 (94.1-99.4)	97 (93.2-99)	97.1 (93.3-99)	97.6 (94-99.3)	0.95
Falcivax	Pure and mixed Pv	76.9 (56.4-91)	100 (98.8-100)	100 (83.2-100)	98.1 (95.9-99.3)	0.86
Onsite Pf/Pv	Pure and mixed Pv	76.9 (56.4-91)	99 (97.2-99.8)	87 (66.4-97.2)	98.1 (95.9-99.3)	0.80
Microscopy	Pure and mixed Pv	76.9 (56.4-91)	99.7 (98.2-100)	95.2 (76.2-99.9)	98.1 (95.8-99.3)	0.84

(PPV = Positive predictive value, NPV = Negative predictive value)

### Rapid diagnostic tests

Of the four RDTs used in this study, Onsite Pf and Paracheck can detect *P. falciparum *only. Sensitivity, specificity, positive predictive value, and negative predictive value for each of the RDTs compared to microscopy (gold standard) and real-time PCR, are given in Table 2 and 3 respectively. Of the RDTs, the highest sensitivities for detection of *P. falciparum *(including mixed infection) were obtained by the Paracheck (98.8%, 95% CI 95.8-99.9) compared to microscopy and Falcivax (97.6%, 95% CI 94.1-99.4) compared to real-time PCR assay respectively. Although Paracheck and Onsite Pf/Pv gave the highest specificity 98.8% (95% CI 95.7-99.9) compared to microscopy and Onsite Pf/Pv 98.8% (95% CI 95.8-99.9) compared to real-time PCR assay respectively. Although both Falcivax and Onsite Pf/Pv had the highest sensitivity (90.5%, 95% CI 69.6-98.8), but Falcivax had the highest specificity (99.7%, 95%CI 98.3-100) compared to microscopy for the detection of *P. vivax*. However, compared to real-time PCR assay, these RDTs gave low sensitivity (76.9%, 95% CI 56.4-91) although very high specificity (100%, 95%CI: 98.8-100) was obtained for 'Falcivax' and for 'Onsite Pf/Pv' (99%, 95% CI: 97.2-99.8) respectively.

## Discussion

Although in Bangladesh, *P. falciparum *and *P. vivax *are the two common prevalent parasites, the majority of malaria cases are caused by *P. falciparum *[1]. However, their ratio varies from time to time. During the nationwide malaria prevalence survey in 2007 based on Falcivax RDT, 90% *P. falciparum *infection was found and the remaining 10% infection was due to pure *P. vivax *or mixed infection of these two parasites although the performance of that RDT (Falcivax) was not evaluated against any gold standard method [3].

This study demonstrated the establishment of pre existing real-time PCR assay modified with SYBR Green dye for the detection of *P. falciparum *and *P. vivax *as an alternative gold standard method for evaluating RDTs used for the diagnosis of malaria. Simultaneously, the performance of microscopy can also be evaluated by this SYBR Green-based PCR method. The original TaqMan-based real-time PCR method was 100% sensitive and specific using nested PCR as gold standard [15]. But in the present study more than 95% of sensitivity and specificity was obtained for both *P. falciparum *and *P. vivax *using microscopy as a gold standard. The modified real-time PCR method detected eight *P. falciparum *and *P. vivax *mixed infections, of which two were detected by microscopy, Falcivax and Onsite Pf/Pv RDT. On the other hand microscopy detected three mixed infections of which modified real-time PCR could detect two and one by Falcivax and Onsite Pf/Pv tests respectively. One sample detected as *P. vivax *by microscopy was detected as mixed infection by modified real-time PCR, Falcivax and Onsite Pf/Pv tests. At the same time one sample was found to be *P. vivax *in all other tests, but detected as *P. falciparum *by microscopy. Two samples were detected negative by microscopy and all the RDTs, but found to have *P. falciparum *in real-time PCR. These two samples were missed in microscopy and RDTs perhaps due to the low number of parasite counts [15,24].

This study was conducted among the symptomatic febrile patients in a high-endemic area. RDTs can play a key role in rapid diagnosis and, hence, prompt treatment of malaria. As RDT can be conducted immediately in the field clinic or even in the field level by the health workers while the patient is present, the most important point for the villagers is the knowledge that they are infected with malaria parasite. On the contrary, the delay in the results of microscopic diagnosis is a serious obstacle for the operation of a malaria control programme in remote areas. Although RDTs have some limitations, all the four tests evaluated had high sensitivity and specificity. The high NPV allow us to confidently diagnose negative test patients as non-malaria patients [25]. Thus, the risk of missing an infected individual is less by the RDTs used in this evaluation. In a similar study in India, high NPV was also recorded for Falcivax RDT [13].

Overall, 55.9% of the febrile patients with suspected malaria in the present study had a positive blood slide, indicating that over half of the suspected cases referred to this hospital (Matiranga UHC) had malaria. A high percentage of malaria cases among the febrile cases of this area could be due to a high prevalence of asymptomatic malaria cases at the community [21].

Pf-HRP 2 based Paracheck is currently being used in the country's NMCP, although there is a necessity of a RDT for detecting multiple malaria infections in the country [1]. In the present study Parachek showed high sensitivity and specificity compared to a study in Malawi where low specificity was reported [26]. However, since the control programme is now targeting for RDTs that can detect multiple infections, this study could provide a valuable guideline to them.

Onsite duo as newly developed test had never been evaluated earlier in any part of the world gave satisfactory results in the present study. Although Onsite Pf/Pv failed to diagnose some *P. falciparum *positive sample which undoubtedly affects its sensitivity compared to its counterpart Falcivax. However, Onsite Pf/Pv gave almost a similar result as like as Falcivax for detecting *P. vivax*.

Low sensitivity of the two RDTs (Falcivax and Onsite Pf/Pv) for the detection of *P. vivax *compared to real-time PCR assay in this present study is similar to studies published earlier [20,26]. This could be due to the inherent limitations of pLDH assay to detect low parasitaemia in the clinical specimens [27]. Miscroscopists similar to RDTs also missed *P. vivax *cases compared to real-time PCR assay perhaps due to the same reason (low parasitaemia)[28].

RDTs do not depend on the operator like microscopy. It was evolved to overcome or reduce the limitations of microscopy. They have brought a revolution in the field of malaria diagnosis. However, these must achieve > 95% sensitivity to prove their usefulness [29]. It has been estimated that over 70 million RDTs are sold across the world. There are a number of companies producing RDTs for the diagnosis of malaria which was initiated by a single company in 1993 [30]. The world health organization listed approximately 50 RDTs, of which only a few had PvLDH antigen-based tests that distinguish between *P. falciparum *and *P. vivax *are commercially available [26].

## Conclusions

Findings of the study suggest that the SYBR Green-based real-time PCR and RDTs used in the study are sensitive and specific for the detection of *P. falciparum*. However, RDTs and microscopy were not sensitive enough compared to real-time PCR assay for the detection of *P. vivax*. The SYBR Green-based real-time PCR could be a useful tool for monitoring the performance of different malaria diagnostic tests in a reference setting by the NMCP. Efforts should be given to increase the accuracy of RDTs as well as microscopy for diagnosis of *P. vivax *in the field level. As more than one malaria parasites are present in the endemic areas of Bangladesh, it is imperative to deploy a RDT that can detect multiple malaria infections by the NMCP.

## Competing interests

The authors declare that they have no competing interests.

## Authors' contributions

MSA conceptualized and designed the study, collected and identified samples, analysed data, drafted the manuscript and made final revisions. MSA, ANM, WAK, NI, MJK, HK, DS, RH did sample analysis and made critical revision of the manuscript. SM organized the field activities, analysed data and helped revise the manuscript. MSA and RH drafted the manuscript. All the authors read the final version of the manuscript and approved.
